# Awareness, practices, and factors of pediatric cardiopulmonary resuscitation among elementary school teachers in Saudi Arabia: A cross-sectional survey

**DOI:** 10.1097/MD.0000000000045365

**Published:** 2025-10-24

**Authors:** Youssef A. Alqahtani, Samy A. Dawood, Ayed A. Shati, Banan S. Alghamdi, Ghayda A. Alghamdi, Saud S. Alasmari, Najla K. Alzahrani, Ebtisam Hamed, Ahmed M. Alhassan, Musab M. Alzahrani, Fai D. Albishri, Syed Esam Mahmood

**Affiliations:** a Department of Child Health, College of Medicine, King Khalid University, Abha, Saudi Arabia; b College of Medicine, Al-Baha University, Al-Baha, Saudi Arabia; c College of Medicine, King Khalid University, Abha, Saudi Arabia; d Faculty of Medicine, Elrazi University, Khartoum, Sudan; e Faculty of Medicine, University of Gezira, Wad Medani, Al Jazirah State, Sudan; f Department of Family and Community Medicine, College of Medicine, King Khalid University, Abha, Saudi Arabia.

**Keywords:** awareness, CPR, elementary school teachers, emergency response, preparedness, Saudi Arabia, training

## Abstract

Cardiopulmonary resuscitation (CPR) is a life-saving technique that is crucial in cases of sudden cardiac arrest. As first responders in many emergencies, elementary school teachers play a critical role in child safety. However, their awareness and preparedness to perform CPR remain underexplored, especially in Saudi Arabia. This study aimed to assess the awareness, practices, and factors influencing CPR preparedness among elementary school teachers in Saudi Arabia. A descriptive cross-sectional study was conducted between May 1, 2024, and October 31, 2024, among 271 elementary school teachers from various regions in Saudi Arabia. The sample was interviewed through a questionnaire-based survey, which included questions on CPR awareness, past training experiences, and attitudes toward CPR. Data were analyzed using SPSS, with a significance level set at *P* < .05. A teacher’s awareness was categorized as “poor” or “good” based on a 60% threshold. The study revealed that a significant proportion of teachers (91.9%) had not studied CPR content, and 83.0% had never received CPR training. While 62.7% of teachers believed that every school should have an automated external defibrillator, only 14.0% reported CPR training as part of the current educational curriculum. Awareness levels regarding CPR were poor among the majority, with 52.0% of teachers unaware of the proper depth and speed of chest compressions. Significant factors influencing awareness included whether teachers were actively studying CPR content (*P* = .030) and their prior training experiences (*P* = .001). Teachers who had received CPR training were more likely to demonstrate better preparedness. The study showed a shortage in CPR training and awareness among elementary school teachers in Saudi Arabia. Despite the majority expressing the need for CPR training, the lack of formal training and consistent educational curricula in CPR poses a challenge. Recommendations include incorporating CPR training into the educational system and ensuring that teachers receive mandatory, accessible, and ongoing CPR education. This will enhance teachers’ readiness to act during emergencies, ensuring better safety for children in schools.

## 1. Introduction

Accidents and sudden illnesses can affect anyone at any time, leaving families and communities vulnerable in moments of crisis.^[1]^ Pediatric cardiac arrest is a highly distressing and complex emergency. Although rare, it carries dire outcomes, with over 90% of out-of-hospital cases resulting in mortality, highlighting the vulnerability of children compared to adults in similar situations.^[2]^ In these critical moments, every second counts. Immediate action, starting with proper CPR, can mean the difference between life and death. High-quality basic life support doesn’t just buy time – it dramatically improves a child’s chances of survival.^[3]^ So far, too many onlookers, including those trusted with children’s safety, lack the knowledge or confidence to act in critical moments. This lack of preparedness is not only a medical concern but also a societal one, highlighting the need for improved education, training, and awareness to safeguard our youngest and most vulnerable.^[4]^

Teachers play a critical role as first responders in school emergencies, including pediatric cardiac arrest, since they are often the first to witness a child collapsing and can initiate life-saving CPR before medical help arrives. Studies show that immediate bystander CPR can double or even triple survival rates in out-of-hospital cardiac arrests among children.^[5]^ However, research in Saudi Arabia indicates that many teachers lack adequate CPR training, with only a few demonstrating sufficient awareness.^[6]^ Mandatory CPR training for educators, as recommended by the American Heart Association, could significantly improve emergency response in schools and save young lives.^[7]^

Literature studies have shown that CPR training greatly improves bystanders’ willingness to perform CPR, making schoolteachers an important group for CPR training initiatives.^[8]^ Teachers are often in situations where they could be the first responders during emergencies, so their readiness and awareness are crucial, especially in cases of cardiac arrest.^[9]^ Several respected organizations support including schoolteachers in CPR training programs to improve bystander CPR rates, recognizing the significant role educators can play in emergency response.^[10]^ The current study aims to assess awareness and preparedness for pediatric cardiopulmonary resuscitation (CPR) among elementary school teachers in Saudi Arabia. Specific objectives include evaluating current knowledge levels, identifying gaps in CPR training, and determining factors influencing readiness to respond to pediatric cardiac arrest incidents.

## 2. Methodology

This study was a descriptive cross-sectional questionnaire-based design aimed at assessing the awareness and practice of pediatric CPR among elementary school teachers in Saudi Arabia. The study was conducted from May 1, 2024, to October 31, 2024, across various elementary schools in Saudi Arabia. This study was approved by the Research Ethical Committee of the College of Medicine, King Khalid University (REC#2024). The sample size was calculated using Epi-Info software (version 7.2.2.6; Centers for Disease Control and Prevention, Atlanta) with a 95% confidence interval, 80% power, and an expected frequency of 50%. The required sample size for the study was determined to be 384 teachers. Given the total population of 597,000 teachers as reported by the Ministry of Education, this sample size was deemed adequate for representing the broader population of elementary school teachers in Saudi Arabia. The questionnaire consisted of several sections, each targeting different aspects of CPR knowledge, attitudes, and practices. The initial section included demographic information. The awareness section of the questionnaire focused on the participants’ knowledge of CPR-related topics, such as the proper steps to take when encountering an unconscious person, the correct technique for chest compressions, and the role of automated external defibrillators (AEDs). It included multiple-choice questions where teachers could select their answers based on their understanding of CPR procedures. The attitude section assessed teachers’ views on whether CPR training should be mandatory in schools, whether it should be included as part of professional licensing, and their beliefs about the presence of AEDs in schools. The practice section aimed to assess whether teachers had ever witnessed CPR being performed or had participated in administering CPR. The final section was designed to explore teachers’ willingness to undergo CPR training, including whether they would sign up for a free course if offered and what barriers or fears they had regarding performing CPR. The questionnaire also explored teachers’ perceptions about the need for CPR training and the availability of training courses. To ensure the validity of the questionnaire, the researchers conducted expert consultations with professionals in emergency medicine, CPR instructors, and education experts. The feedback from these consultations helped refine the wording, structure, and relevance of the questions. Additionally, the questionnaire was subjected to a pilot study with 15 teachers to assess its reliability. The internal consistency of the tool was evaluated using Cronbach Alpha, which yielded a coefficient of 0.72, indicating that the instrument was reliable for use in the main study.

Data were collected via an online questionnaire, distributed through various channels such as educational institutions, community centers, and social media platforms. Electronic informed consent was obtained from all the participants before filling out the survey forms. The questionnaire was made accessible through a Google Form, which facilitated easy participation. To be eligible for inclusion, participants were required to be Saudi citizens, elementary school teachers, and to provide informed consent. Exclusion criteria included non-Saudis, non-elementary school teachers, and those who did not complete the questionnaire. The recruitment process ran for 6 months, from May to October 2024, with continuous promotion of the survey to maximize participation. Participants were recruited through various platforms, including social media, flyers, and emails. Interested teachers were directed to the online platform to provide consent and complete the survey. Participation was voluntary, and participants had the option to withdraw at any point without penalty. Data collection commenced once informed consent was obtained from all willing participants.

### 2.1. Data analysis

The data analysis for the study was conducted using SPSS version 27 (IBM Corp., Armonk, 2020). The overall awareness level of pediatric CPR among teachers was calculated by assigning 1 point for each correct answer, and teachers with an overall score of <60% were classified as having poor awareness, while those with a score % exceeding 60% were considered to have a good awareness level. Frequencies and percentages were used to describe categorical variables, such as region, age, gender, marital status, and prior CPR training experience. Factors associated with awareness and practice were analyzed using Pearson Chi-square (χ^2^) tests and Exact Probability tests as appropriate, with a significance level set at *P* < .05. These methods were used to assess the relationship between various factors, such as region, age, gender, marital status, training history, and participation in CPR, with awareness and practice of CPR.

## 3. Results

Table 1 presents the socio-demographic characteristics of 271 elementary school teachers in Saudi Arabia. In terms of region, the majority of teachers (178 or 65.7%) are from the Southern Region, followed by 42 teachers (15.5%) from the Western Region, 32 teachers (11.8%) from the Central Region, and 19 teachers (7.0%) from the Eastern Region. The largest age group is 40 to 49 years, comprising 151 teachers (55.7%), while 60 teachers (22.1%) fall into both the 20 to 39 and 50 to 59 age groups. As for gender, there are 113 male teachers (41.7%). Regarding marital status, most teachers are married (250 or 92.3%), with only 21 teachers (7.7%) being single. About CPR content studies, 22 teachers (8.1%) are currently studying CPR. Looking at the previous CPR training, only 46 teachers (17.0%) have received such training. Among those who have been trained, the majority (19 teachers or 41.3%) received their training at school, followed by 16 teachers (34.8%) who were trained by Nonprofit organization. Regarding the duration since the training, the majority (28 teachers or 60.9%) received CPR training more than 24 months ago, while 10 teachers (21.7%) were trained within the past 6 months, 4 teachers (8.7%) were trained between 7 to 12 months ago, and another 4 teachers (8.7%) received training between 13 and 24 months ago. Finally, considering the reasons for not receiving CPR training, the most common reason is lack of time, reported by 92 teachers (40.9%), followed by a lack of interest (45 teachers or 20.0%), uncertainty about where to take the course (44 teachers or 19.6%), and course fees (17 teachers or 7.6%). Fewer teachers cited a lack of available courses (14 teachers or 6.2%) and a lack of knowledge about such courses (9 teachers or 4.0%).

**Table 1:** Socio-demographic characteristics of the study elementary school teachers, Saudi Arabia (N = 271).

Socio-demographics	No	%
Region		
Central Region	32	11.8
Eastern Region	19	7.0
Western Region	42	15.5
Southern Region	178	65.7
Age (yr)		
20–39	60	22.1
40–49	151	55.7
50–59	60	22.1
Gender		
Male	113	41.7
Female	158	58.3
Marital status		
Single	21	7.7
Married	250	92.3
Are you studying CPR content?		
Yes	22	8.1
No	249	91.9
Have you previously received a CPR training course?		
Yes	46	17.0
No	225	83.0
Site of received training		
At University	1	2.2
Nonprofit organization	16	34.8
At school	19	41.3
Private organization	3	6.5
Others	7	15.2
Duration since training		
0–6 mo	10	21.7
7–12 mo	4	8.7
13–24 mo	4	8.7
>24 mo	28	60.9
Reasons for not having CPR training		
No right time to do	92	40.9
Not interested	45	20.0
Not sure where to join the course	44	19.6
Due to course fees and costs	17	7.6
No available courses	14	6.2
Lack of knowledge about these courses	9	4.0
Others	4	1.8

CPR = cardiopulmonary resuscitation.

Table 2 provides the awareness of Pediatric CPR among elementary school teachers in Saudi Arabia. When asked what to do if encountering an unconscious person alone, the majority of teachers (184 or 67.9%) correctly identified the steps to check consciousness, secure the airway, and check if the patient is breathing. Regarding what to do if the patient is breathing but unresponsive to verbal stimulation, 138 teachers (50.9%) correctly stated that the patient should be placed in the lateral prone position and an ambulance should be called. In terms of CPR procedures, the correct sequence of 30 chest compressions followed by 2 rescue breaths was identified by 69 teachers (25.5%), while a significant proportion of teachers (99 or 36.5%) were unsure. For the proper depth and speed of chest compressions, 58 teachers (21.4%) knew that the compressions should be at least 5 cm deep and at a rate of 100 to 120 per minute. When it comes to the purpose of an AED, 153 teachers (56.5%) correctly recognized that the device analyzes heart rhythm disturbances and administers an electric shock if necessary. As for who is allowed to use an AED, 204 teachers (75.3%) correctly indicated that only qualified/trained persons should use it. In a practical scenario where the teacher is the first responder, 209 teachers (77.1%) knew that they should kneel near the chest of the injured person. Lastly, when asked about the correct hand position for chest compressions, 175 teachers (64.6%) correctly identified that one should place one hand on top of the other with the palm facing down.

**Table 2:** Awareness of pediatric cardiopulmonary resuscitation among elementary school teachers in Saudi Arabia (N = 271).

Items	No	%
If you are alone and encounter an unconscious person, what do you do?		
Check consciousness, secure the airway, and check if the patient is breathing	184	67.9
Check the pulse	51	18.8
Start chest compressions immediately	9	3.3
I don’t know	27	10.0
If the patient is found to be breathing, but does not respond to verbal stimulation (calling loudly), what do you do?		
Place the patient in the lateral prone position and call an ambulance	138	50.9
Check the pulse	40	14.8
Start chest compressions immediately	55	20.3
I don’t know	38	14.0
You have decided to perform CPR. Which of the following actions, and in what order, will you choose?		
30 chest compressions: 2 rescue breaths	69	25.5
30 chest compressions: 5 rescue breaths	63	23.2
2 rescue breaths: 30 chest compressions	40	14.8
I don’t know	99	36.5
How deep and fast should chest compressions be?		
At least 5 cm and 100–120/min	58	21.4
5–4 cm and 100/min	72	26.6
I don’t know	141	52.0
What is the purpose of an automated external defibrillator (AED)?		
Analyze heart rhythm disturbances and administer an electric shock if necessary	153	56.5
Giving a heart massage	54	19.9
Heart rhythm disturbance analysis	9	3.3
I don’t know	55	20.3
Who is allowed to use an automated external defibrillator (AED)?		
Only qualified/trained persons should use it	204	75.3
Emergency department staff only	28	10.3
Everyone	9	3.3
I don’t know	30	11.1
If you were the first responder to the injured person, would you kneel near the chest?		
Yes	209	77.1
No	16	5.9
I don’t know	46	17.0
What is the correct hand position for chest compressions?		
Use your hands by placing one hand on top of the other with the palm facing down	175	64.6
Use your hands with your fingers interlaced	45	16.6
I use my hands side by side	13	4.8
I don’t know	38	14.0

CPR = cardiopulmonary resuscitation.

**Figure 1.**
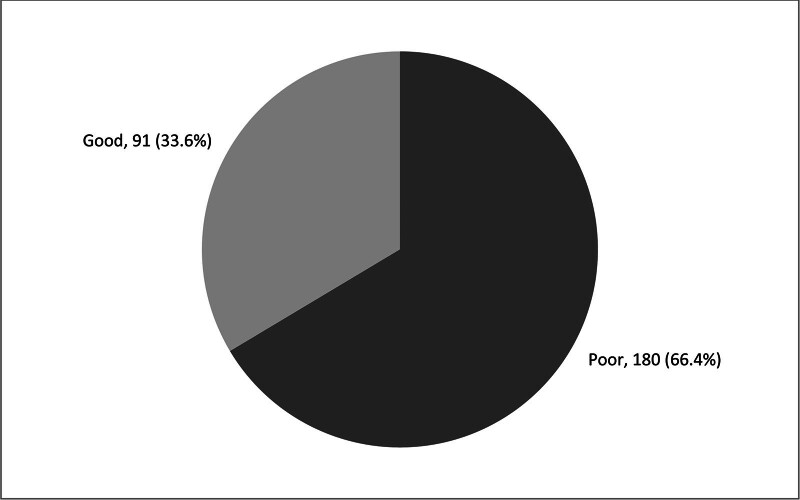
The overall awareness of pediatric cardiopulmonary resuscitation among elementary school teachers in Saudi Arabia (N = 271).

Figure 1 shows the overall awareness of Pediatric CPR among elementary school teachers in Saudi Arabia. A significant portion of the teachers, 180 out of 271 (66.4%), have a poor level of awareness regarding pediatric CPR. In contrast, 91 teachers (33.6%) demonstrate a good level of awareness.

Table 3 assesses the attitudes of elementary school teachers in Saudi Arabia towards Pediatric CPR. Regarding the mandatory nature of CPR training, opinions are divided. While 115 teachers (42.4%) believe CPR training should be mandatory in schools, 71 teachers (26.2%) think it should be required for every job. However, a significant portion, 81 teachers (29.9%), feel that CPR training should be optional. Only 4 teachers (1.5%) believe CPR training should be required to obtain a driving license. Concerning whether CPR training is part of the current educational curriculum, 126 teachers (46.5%) responded negatively, stating that it is not included, while 38 teachers (14.0%) reported it is part of the curriculum. A large group of 107 teachers (39.5%) were unsure about this matter. When asked about the requirement for a professional instructor license, 64 teachers (23.6%) agreed that CPR training should be a requirement, while 207 teachers (76.4%) disagreed. Finally, regarding the presence of an AED in schools, a majority of 170 teachers (62.7%) believe that every school should have an AED, while 101 teachers (37.3%) disagree.

**Table 3:** Attitude towards pediatric cardiopulmonary resuscitation among elementary school teachers in Saudi Arabia (N = 271).

Attitude	No	%
Do you think CPR training should be mandatory?		
Yes, in schools	115	42.4
Yes, to obtain a driving license	4	1.5
Yes, training should be mandatory in every job	71	26.2
No, CPR training should be optional	81	29.9
Is CPR training part of the current educational curriculum?		
Yes	38	14.0
No	126	46.5
I don’t know	107	39.5
Do you think CPR training should be a requirement for a professional instructor license?		
Yes	64	23.6
No	207	76.4
Do you think every school should have an automated external defibrillator (AED)?		
Yes	170	62.7
No	101	37.3

CPR = cardiopulmonary resuscitation.

As for preparedness to perform CPR (Table 4), the primary reason people are afraid to perform CPR on injured individuals is a lack of proper knowledge and skills, with 133 teachers (49.1%) selecting this option. Fear of causing harm to the injured person is another significant concern, chosen by 101 teachers (37.3%). Fewer teachers cited fear of contracting infectious diseases through mouth-to-mouth breathing (21 teachers, 7.7%), emotional factors (14 teachers, 5.2%), or fear of legal consequences (2 teachers, 0.7%) as reasons for hesitancy. Considering interest in training, a large majority of teachers expressed a desire to receive CPR training, with 241 teachers (88.9%) indicating they would like to take a course. Additionally, when offered a free CPR training course, an even higher number of teachers (247, or 91.1%) said they would be willing to sign up.

**Table 4:** Preparedness to perform CPR and interest in CPR training among elementary school teachers in Saudi Arabia (N = 271).

Items	No	%
Why are people afraid to perform CPR on injured people?		
Lack of proper knowledge and skills	133	49.1
Fear of causing harm to the injured person	101	37.3
Fear of contracting infectious diseases through mouth-to-mouth breathing	21	7.7
Emotional factors	14	5.2
Fear of legal consequences	2	.7
Do you want a CPR training course?		
Yes	241	88.9
No	30	11.1
If a free CPR training course were offered, would you be willing to sign up for it?		
Yes	247	91.1
No	24	8.9

CPR = cardiopulmonary resuscitation.

**Figure 2.**
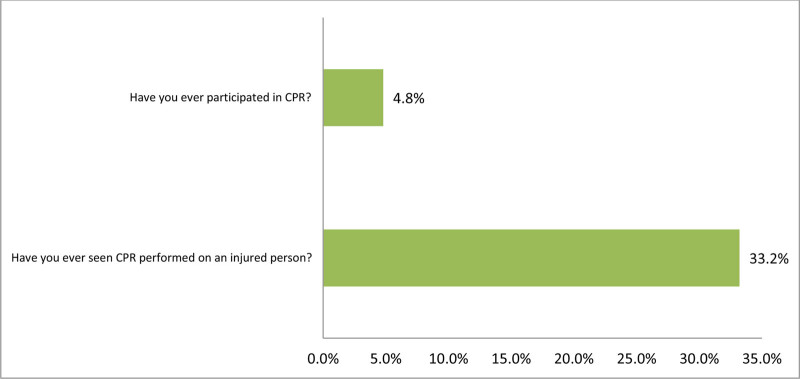
Elementary school teachers’ practice of CPR in Saudi Arabia (N = 271). CPR = cardiopulmonary resuscitation.

Figure 2 provides the practice of CPR among elementary school teachers in Saudi Arabia. Exact of 90 teachers (33.2%) have witnessed CPR being performed on an injured person. However, only 13 teachers (4.8%) have participated in performing CPR themselves.

Table 5 examines the factors associated with elementary school teachers’ awareness of Pediatric PCR in Saudi Arabia. A significant factor influencing awareness is the region (*P*-value = 0.049). Teachers from the Southern Region showed the highest proportion of good awareness (30.3%) compared to those from other regions, while teachers from the Eastern Region had the lowest proportion of good awareness (26.3%). Another significant factor is whether teachers are currently studying CPR content (*P*-value = .030). Among those studying CPR, 54.5% had good awareness, compared to only 31.7% of those not studying CPR. Other factors, such as age, gender, marital status, CPR training, site of training, duration since training, and previous experiences with seeing or participating in CPR, did not show statistically significant associations with awareness (*P*-values ranging from .156–.826).

**Table 5:** Factors associated with teachers awareness about PCR in Saudi Arabia.

Factors	Overall awareness level				*P*-value
	Poor		Good		
	No	%	No	%	
Region					
Central Region	15	46.9	17	53.1	.049*,†
Eastern Region	14	73.7	5	26.3	
Western Region	27	64.3	15	35.7	
Southern Region	124	69.7	54	30.3	
Age (yr)					
20–39	36	60.0	24	40.0	.394
40–49	101	66.9	50	33.1	
50–59	43	71.7	17	28.3	
Gender					
Male	75	66.4	38	33.6	.988
Female	105	66.5	53	33.5	
Marital status					
Single	11	52.4	10	47.6	.156
Married	169	67.6	81	32.4	
Are you studying CPR content?					
Yes	10	45.5	12	54.5	.030*
No	170	68.3	79	31.7	
Have you previously received a CPR training course?					
Yes	28	60.9	18	39.1	.382
No	152	67.6	73	32.4	
Site of received training					
At university	0	0.0	1	100.0	.309†
Hilal Al-Ahmar	10	62.5	6	37.5	
At school	14	73.7	5	26.3	
Private organization	1	33.3	2	66.7	
Others	3	42.9	4	57.1	
Duration since training					
0–6 mo	8	80.0	2	20.0	.553†
7–12 mo	2	50.0	2	50.0	
13–24 mo	2	50.0	2	50.0	
>24 mo	16	57.1	12	42.9	
Have you ever seen CPR performed on an injured person?					
Yes	61	67.8	29	32.2	.739
No	119	65.7	62	34.3	
Have you ever participated in CPR?					
Yes	9	69.2	4	30.8	.826
No	171	66.3	87	33.7	

*P* = Pearson χ^2^ test, CPR = cardiopulmonary resuscitation.

* *P* < .05 (significant).

† Exact probability test.

Table 6 explores factors associated with elementary school teachers’ participation in Pediatric PCR in Saudi Arabia. Significant factors influencing participation in CPR include whether teachers are currently studying CPR content (*P*-value = .043) and whether they have previously received CPR training (*P*-value = .001). Among those who are studying CPR content, 13.6% (3 teachers) reported having participated in CPR, compared to only 4.0% (10 teachers) of those not studying CPR. Similarly, teachers who had previously received a CPR training course had a higher rate of participation in CPR (21.7% or 10 teachers) compared to those without any CPR training (1.3% or 3 teachers). Other factors such as region, age, gender, marital status, site of training, and duration since training did not show statistically significant associations with participation in CPR (*P*-values ranging from .263–.994).

**Table 6:** Factors associated with teachers’ practice of PCR in Saudi Arabia.

Factors	Have you ever participated in CPR?				*P*-value
	Yes		No		
	No	%	No	%	
Region					
Central Region	0	0.0	32	100.0	.597†
Eastern Region	1	5.3	18	94.7	
Western Region	2	4.8	40	95.2	
Southern Region	10	5.6	168	94.4	
Age (yr)					
20–39	1	1.7	59	98.3	.263†
40–49	10	6.6	141	93.4	
50–59	2	3.3	58	96.7	
Gender					
Male	4	3.5	109	96.5	.413
Female	9	5.7	149	94.3	
Marital status					
Single	1	4.8	20	95.2	.994
Married	12	4.8	238	95.2	
Are you studying CPR content?					
Yes	3	13.6	19	86.4	.043*
No	10	4.0	239	96.0	
Have you previously received a CPR training course?					
Yes	10	21.7	36	78.3	.001*
No	3	1.3	222	98.7	
Site of received training					
At university	0	0.0	1	100.0	.783†
Hilal Al-Ahmar	2	12.5	14	87.5	
At school	5	26.3	14	73.7	
Private organization	1	33.3	2	66.7	
Others	2	28.6	5	71.4	
Duration since training					
0–6 mo	3	30.0	7	70.0	.303†
7–12 mo	2	50.0	2	50.0	
13–24 mo	0	0.0	4	100.0	
>24 mo	5	17.9	23	82.1	

*P* = Pearson χ^2^ test, CPR = cardiopulmonary resuscitation.

* *P* < .05 (significant).

† Exact probability test.

## 4. Discussion

Elementary school teachers are often the first adults on the scene when a child has a medical emergency, so knowing CPR is crucial. This study assessed Saudi Arabian elementary school teachers’ knowledge of pediatric CPR and found varying levels of awareness.

While many teachers understand the basics, like checking for consciousness and knowing what an AED is, they struggle with the specifics of how to perform CPR correctly, such as compression depth and rate. This isn’t just a problem in Saudi Arabia; similar issues have been seen worldwide.^[11–13]^ Our study findings reveal a pattern of stronger awareness in initial assessment procedures but weaker understanding of technical CPR execution details. Most teachers had good knowledge regarding initial response to an unconscious person, with a significant majority correctly identifying the proper steps to check consciousness, secure the airway, and assess breathing. Similarly, teachers showed reasonable awareness about managing unresponsive but breathing patients, with approximately half correctly identifying the recovery position as appropriate in such scenarios. The awareness level regarding AED usage was particularly encouraging, with most teachers correctly understanding the purpose of this device in analyzing heart rhythm disturbances and delivering electric shocks when necessary. Additionally, teachers demonstrated good awareness of practical positioning aspects of CPR, with most correctly identifying the proper position for a first responder and the correct hand placement for chest compressions.

However, regarding technical knowledge of CPR, only about a quarter of teachers correctly identified the proper sequence of chest compressions and rescue breaths, while an even smaller proportion demonstrated accurate knowledge of the correct depth and rate of compressions.

This discrepancy between conceptual understanding and technical knowledge suggests that while teachers may grasp the importance and general principles of CPR, they lack the specific procedural knowledge required for effective implementation. The significant proportion of teachers who expressed uncertainty about proper CPR procedures further underscores this knowledge deficit. These findings are consistent with the broader literature on CPR awareness among non-healthcare professionals, where conceptual understanding often exceeds procedural knowledge.^[11,14,15]^

When comparing our findings with other studies conducted in Saudi Arabia, several notable patterns emerge that provide background for understanding CPR awareness among elementary school teachers nationally. The study by Alahmed and colleagues in the Qassim region revealed that only a small fraction of school teachers had received previous CPR training, with the majority of those trained having completed their training more than 2 years before the study.^[16]^ This matches our findings, where technical knowledge of CPR procedures was limited, suggesting a widespread lack of formal training or significant skill decay over time among Saudi teachers. The Qassim study further identified that the primary barrier to applying basic life support was the lack of proper knowledge and skills, reported by nearly half of the teachers surveyed. This corresponds with our observation that while teachers recognize the importance of CPR, many lack confidence in the technical aspects of its execution. Interestingly, the Qassim study found that a substantial majority of teachers expressed a desire for more CPR training, indicating a positive attitude toward improving their resuscitation skills despite current knowledge deficits. Similarly, the study by Alharbi and colleagues in Riyadh found that more than half of school teachers had no previous information about CPR, while less than half reported some knowledge.^[17]^ This closely reflects the pattern in our study, where awareness of basic concepts was present but detailed procedural knowledge was lacking. The Riyadh study also revealed varied sources of CPR information among teachers, including university education, training courses, television, and the internet. This variety of information sources may explain the inconsistent knowledge levels observed in our study, as formal training was relatively uncommon. A particularly concerning finding from the Riyadh study was that only about two-thirds of teachers knew the contact numbers for emergency services, highlighting a fundamental gap in emergency response knowledge. While our study did not specifically assess this aspect, the overall pattern of incomplete emergency response knowledge appears consistent across studies. The Riyadh researchers also found that approximately half of the teachers agreed that CPR training courses should be mandatory, suggesting moderate support for formalized training requirements. Both local studies highlighted the critical need for mandatory implementation of CPR and basic life-support training for all school teachers across Saudi Arabia. This recommendation is strongly supported by our findings, particularly given the knowledge gaps in the technical aspects of CPR performance. The consistency of findings across different regions of Saudi Arabia suggests that CPR knowledge deficiencies among school teachers represent a national challenge rather than a regional issue, requiring coordinated policy responses at the national level.

Examining our findings within a global context reveals both similarities and differences in CPR awareness patterns among school teachers internationally. The study by Alwidyan and colleagues in Jordan provides an important regional comparison from another Middle Eastern country. Their research found that only a small percentage of Jordanian schoolteachers had received CPR training, which is comparable to the low rates of technical CPR knowledge observed in our study and other Saudi Arabian research.^[18]^ Interestingly, the Jordanian study revealed that teachers with previous CPR training had significantly higher knowledge scores, indicating the critical importance of formal training in developing CPR competence. Looking beyond the Middle East, the Danish study by Hansen and colleagues offers valuable insights from a country with a longer history of mandated CPR training in schools. Despite 8 years of legislative mandates in Denmark, their nationwide survey found that only about a quarter of eligible classes had completed CPR training, and only a tenth had received AED training.^[19]^ This surprisingly low implementation rate in a developed country with established legislation suggests that mandating CPR training alone is insufficient without addressing implementation barriers. International studies from other regions, while not examined in detail here, generally support the pattern of inadequate CPR knowledge among school teachers globally. Research from various countries consistently identifies poor knowledge in technical aspects of CPR performance, highlighting the universal challenge of preparing teachers to respond effectively to cardiac emergencies in school settings.^[20,21]^

Generally, our study found that a large majority of elementary school teachers have poor knowledge of pediatric CPR. Similar previous research in the region showed similar findings with low CPR knowledge among teachers.^[22,23]^ This lack of preparedness may be due to the absence of mandatory CPR training in Saudi schools, unlike some Western countries.^[24]^ Since teachers are often the first responders in child emergencies, improving their CPR skills is essential. Targeted training programs have been shown to increase CPR knowledge and confidence.^[25]^

Regarding preparedness, our study showed a clear need for CPR training among elementary school teachers. The biggest barrier to performing CPR is a lack of knowledge and skills, followed by fear of causing harm. Despite these concerns, there’s a strong interest in receiving training, with over 88% of teachers wanting to take a CPR course, and even more willing to sign up if it’s free. This suggests that providing accessible, effective training could significantly improve teachers’ CPR preparedness.

### 4.1. Strength and limitation

The study focuses on a critical public health issue. The study addresses an important gap in understanding the level of CPR awareness among teachers responsible for child safety, which can inform targeted training programs and policy development. One notable limitation of this study is the reliance on self-reported data collected through an online questionnaire, which may be subject to response bias and social desirability bias. Participants might overestimate their knowledge or practice of CPR or provide responses they perceive as favorable rather than reflecting their actual awareness and experiences. To mitigate these biases, the questionnaire was carefully designed to ensure anonymity and confidentiality, encouraging honest responses. Additionally, clear instructions emphasized that there were no right or wrong answers and that honest self-assessment was essential for the accuracy of the study. Despite these measures, some degree of bias may still be present, and these limitations were acknowledged as inherent to the study design.

Additionally, the use of an online distribution method may have limited participation to teachers with easy internet access and familiarity with digital platforms, potentially affecting the representativeness of the sample. Furthermore, despite expert validation and a pilot test, the questionnaire’s ability to accurately measure actual CPR competence rather than perceived knowledge remains limited. These factors could influence the generalizability and accuracy of the findings regarding teachers’ true levels of CPR awareness and practice.

### 4.2. Conclusions and recommendations

The study revealed poor awareness and practice of pediatric CPR among elementary school teachers in Saudi Arabia. While many teachers expressed a willingness to receive CPR training, a large proportion lacked the necessary knowledge to perform CPR effectively in emergencies. Factors such as prior CPR training and regional differences influenced awareness levels, with teachers from the Southern Region demonstrating better knowledge. Despite the recognition of CPR’s importance, most teachers had not participated in or witnessed CPR in real-life situations. Based on these findings, it is recommended that CPR training become mandatory for all elementary school teachers, with efforts to overcome barriers like time, cost, and awareness of available courses. Additionally, schools should ensure the availability of AEDs and focus on practical, scenario-based training to enhance teachers’ preparedness to respond to emergencies.

## Acknowledgments

The authors extend their appreciation to the Deanship of Research and Graduate Studies at King Khalid University for funding this work through Small Research Project under grant number RGP1/100/46.

## Author contributions

**Conceptualization:** Youssef A. Alqahtani.

**Data curation:** Banan S. Alghamdi, Ghayda A. Alghamdi, Saud S. Alasmari, Najla K. Alzahrani, Musab M. Alzahrani, Fai D. Albishri.

**Investigation:** Youssef A. Alqahtani, Samy A. Dawood, Banan S. Alghamdi, Ghayda A. Alghamdi, Saud S. Alasmari, Najla K. Alzahrani, Musab M. Alzahrani, Fai D. Albishri.

**Methodology:** Youssef A. Alqahtani, Syed Esam Mahmood.

**Project administration:** Ayed A. Shati.

**Resources:** Ebtisam Hamed.

**Supervision:** Ayed A. Shati.

**Validation:** Ayed A. Shati, Ebtisam Hamed, Ahmed M. Alhassan, Fai D. Albishri, Syed Esam Mahmood.

**Visualization:** Samy A. Dawood, Ayed A. Shati.

**Writing – original draft:** Youssef A. Alqahtani.

**Writing – review & editing:** Samy A. Dawood, Ayed A. Shati, Ebtisam Hamed, Ahmed M. Alhassan, Syed Esam Mahmood.
